# Deciphering hub genes and immune landscapes related to neutrophil extracellular traps in spinal cord injury: insights from integrated bioinformatics analyses and experiments

**DOI:** 10.3389/fimmu.2026.1742155

**Published:** 2026-03-05

**Authors:** Xiaoqin Liu, Jiating Hu, Chunxia Liu, Guodong Shi, Wenxia Zhu, Xuan Zhou

**Affiliations:** 1 Yan’an Medical College of Yan’an University, Yan’an, China; 2 Department of Radiology, The Affiliated Hospital of Yan’an University, Yan’an, China; 3 Department of Neurosurgery, The Affiliated Hospital of Yan’an University, Yan’an, China

**Keywords:** bioinformatics, immune infiltration, machine learning, neutrophil extracellular traps, spinal cord injury

## Abstract

**Background:**

Spinal cord injury (SCI) is a debilitating neurological condition that results in severe motor, sensory, and autonomic dysfunction, imposing a considerable burden on affected individuals and healthcare systems. Neutrophil extracellular traps (NETs) have been increasingly implicated in inflammatory and immune responses; however, the roles of NETs-related genes (NRGs) in SCI remain poorly understood. This study aimed to investigate the involvement of NRGs in SCI pathophysiology and to identify NET-associated candidate genes of potential biological relevance.

**Methods:**

The GSE151371 dataset was obtained from the Gene Expression Omnibus (GEO) to identify NRGs associated with SCI. Differential expression analysis and weighted gene co-expression network analysis (WGCNA) were performed to screen candidate genes, followed by machine learning algorithms for hub gene prioritization. The identified hub genes were validated using an independent dataset (GSE45006). Immune cell composition in peripheral blood samples was estimated using the CIBERSORT algorithm based on a predefined leukocyte gene signature matrix. In addition, the expression of the hub gene was validated in a rat SCI model using RT-qPCR and immunofluorescence.

**Results:**

We identified ten intersecting genes as candidate differentially expressed NRGs in SCI. After prioritization of hub genes using multiple machine learning algorithms, FCGR1A, CLEC6A, and RETN were identified. Subsequent validation in the independent dataset GSE45006 demonstrated that only FCGR1A showed significant differential expression. In SCI samples, FCGR1A expression showed a positive correlation with activated mast cells and naïve CD4^+^ T cells, while exhibiting a negative correlation with naïve B cells and resting memory CD4^+^ T cells. Moreover, *in vivo* experiments confirmed the upregulation of FCGR1A at both the mRNA and protein levels in SCI models, supporting its association with SCI-related inflammatory responses.

**Conclusions:**

This study provides integrative bioinformatics and experimental evidence supporting the involvement of NETs-related genes in SCI and identifies FCGR1A as a NET-associated biomarker candidate linked to immune and inflammatory responses in SCI, warranting further mechanistic investigation.

## 1 Introduction

Spinal cord injury (SCI) is a debilitating neurological condition characterized by severe motor, sensory, and autonomic dysfunction resulting from damage to the spinal cord (1, 2). The incidence and prevalence of SCI are increasing worldwide, leading to substantial morbidity and imposing significant healthcare costs and societal burdens due to long-term disability and rehabilitation requirements (3, 4). Despite advances in clinical management, including hemodynamic support, early decompressive surgery, and therapeutic hypothermia, effective strategies to halt disease progression or reverse neurological deficits remain limited (5). Secondary injury processes, particularly neuroinflammation, play a central role in the progression of SCI pathology. Because the primary mechanical injury is largely irreversible, therapeutic efforts have increasingly focused on targeting the subsequent inflammatory cascade to mitigate secondary damage and improve functional outcomes (6). Neuroinflammation is initiated by disruption of the blood–spinal cord barrier (BSCB), which permits the infiltration of peripheral immune cells, including neutrophils, monocytes, and lymphocytes, into the injured spinal cord (7). These infiltrating immune cells release proinflammatory cytokines, chemokines, reactive oxygen species (ROS), and nitric oxide, thereby amplifying local inflammation and exacerbating secondary tissue damage (8). Among these immune cells, neutrophils have emerged as key contributors to secondary injury following SCI (9). Although their precise role remains debated, accumulating evidence suggests that activated neutrophils exacerbate tissue damage through the release of cytotoxic mediators such as ROS and pro-inflammatory cytokines, including TNF-α, IL-1β, and IL-6 (9, 10). In addition, activated neutrophils can generate neutrophil extracellular traps (NETs), which are web-like structures composed of extracellular DNA and granular proteins. Recent studies have highlighted the involvement of NETs in driving inflammation and tissue injury after SCI, underscoring their potential relevance to disease progression (7, 11). The emerging association between NETs and SCI suggests that targeting NET formation or NET-related pathways may represent a promising therapeutic strategy. In parallel, an increasing number of neutrophil extracellular trap–related genes (NRGs) have been identified across various inflammatory and immune-mediated diseases (12, 13). However, the comprehensive landscape of NRGs and their functional relevance in SCI remain poorly understood. Therefore, the present study aimed to systematically characterize NET-related gene signatures in SCI and to explore their potential roles in disease pathophysiology.

**Figure 1:**
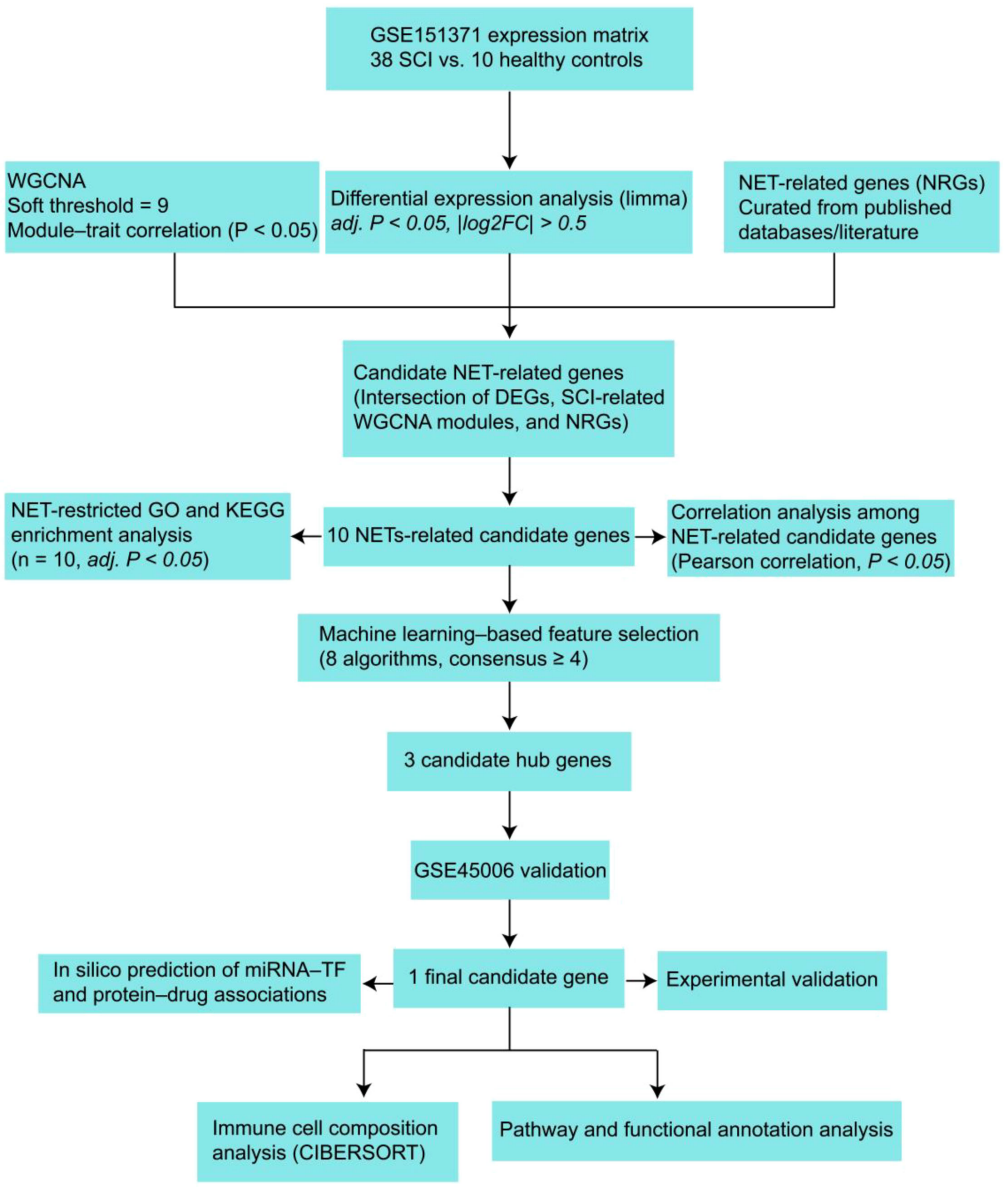
Workflow for identifying NET-related molecular mechanisms in SCI. All downstream functional, correlation, and predictive analyses were performed on pre-selected NET-related gene subsets and should be interpreted as correlation-based and hypothesis-generating.

To achieve this, we analyzed gene expression profiles from the GEO database. Weighted gene co-expression network analysis (WGCNA) and differential expression analysis were applied to the GSE151371 dataset to identify SCI-associated gene modules and differentially expressed genes (DEGs). By intersecting these results with a curated list of NRGs derived from the literature, we identified 10 differentially expressed NRGs (DE-NRGs). Machine learning algorithms were subsequently used to prioritize hub genes, among which only FCGR1A was consistently validated in an independent dataset (GSE45006). We further performed immune infiltration analysis to investigate the association between FCGR1A and immune cell dynamics in SCI. Finally, the dysregulated expression of FCGR1A was validated in a rat SCI model, providing experimental support for its potential involvement in SCI-associated immune responses. An overview of the study design is presented in **Figure 1**.

## 2 Materials and methods

### 2.1 Dataset and preprocessing

The present study utilized the GSE151371 dataset obtained from the Gene Expression Omnibus (GEO) database, which contains peripheral blood RNA expression profiles from 38 patients with SCI and 10 healthy controls (HC). In addition, the GSE45006 dataset, generated using the Affymetrix Rat Genome 230 2.0 Array (GPL1355), was included for experimental validation. This dataset comprises 4 sham controls and 20 SCI samples collected at 1, 3, 7, 14, and 56 days post-injury (n = 4 per time point). For the present analysis, only samples collected during the acute phase of SCI (≤7 days post-injury) were included.

All transcriptomic analyses, including differential expression analysis and weighted gene co-expression network analysis (WGCNA), were initially performed on the full expression matrix. A curated list of 231 neutrophil extracellular trap (NET)-related genes was subsequently obtained from published literature and public databases (**Supplementary Table 1**) (14–19). These NET-related genes were intersected with downstream analytical results to focus on NET-associated molecular mechanisms involved in SCI.

### 2.2 Weighted correlation network analysis

WGCNA was conducted on the full expression matrix of the GSE151371 dataset using the “WGCNA” R package (version 1.72-1) (20). Prior to network construction, hierarchical clustering was performed to detect and remove potential outlier samples based on sample connectivity. Genes with low expression variance were filtered to reduce noise. A scale-free co-expression network was constructed by selecting an appropriate soft-thresholding power (β) using the pickSoftThreshold function according to the scale-free topology criterion. The adjacency matrix was transformed into a topological overlap matrix (TOM), and hierarchical clustering was performed to identify gene modules. Modules were detected using the dynamic tree-cut algorithm with a minimum module size of 50 genes. Gene significance (GS) was defined as the Pearson correlation between individual gene expression levels and the SCI trait. GS values, together with module membership (MM), were used to evaluate the biological relevance of genes within SCI-associated modules. No strict GS cutoff was applied; instead, GS–MM relationships were visualized to assess the overall association between gene modules and SCI.

### 2.3 Identification of differentially expressed genes

Differential expression analysis was performed on the full GSE151371 dataset to identify DEGs between SCI and HC samples using the ‘limma’ R package (version 3.62.2) (21). Raw *P*-values were adjusted for multiple testing using the Benjamini–Hochberg false discovery rate (FDR) method. Genes with an adjusted *P*-value < 0.05 and an absolute log2 fold change (|log2FC|) > 0.5 were considered significantly differentially expressed. Volcano plots were generated using the “ggplot2” R package (version 3.5.2).

### 2.4 Gene ontology and Kyoto encyclopedia of genes and genomes pathways functional enrichment analysis

GO and KEGG enrichment analyses were conducted using the ‘clusterProfiler’ R package (version 4.14.4) (22) to explore the biological functions and molecular pathways associated with DE-NRGs. GO enrichment analysis included the Biological Process (BP), Cellular Component (CC), and Molecular Function (MF) categories. Enrichment significance was evaluated using a hypergeometric test, and multiple-testing correction was applied using the Benjamini–Hochberg false discovery rate (FDR) method. GO terms and KEGG pathways with an adjusted *P*-value < 0.05 and q-value < 0.1 were considered significantly enriched. Gene identifiers were converted to gene symbols for result interpretation and visualization.

### 2.5 Identification of candidate hub genes using machine learning algorithms

Machine learning–based feature selection was employed to identify candidate hub genes associated with NET-related mechanisms in SCI. The input features for machine learning analyses consisted of genes derived from the intersection of DEGs, SCI-associated WGCNA modules, and curated NET-related genes. Eight machine learning algorithms were applied, including Bagged Trees, Bayesian models, Random Forest (RF), Boruta, Learning Vector Quantization (LVQ), Least Absolute Shrinkage and Selection Operator (LASSO), Support Vector Machine (SVM), and eXtreme Gradient Boosting (XGBoost) (23). All analyses were performed using the ‘caret’, ‘glmnet’, ‘Boruta’, and ‘xgboost’ R packages. For SVM, XGBoost, Random Forest, Bagged Trees, and Bayesian models, recursive feature elimination (RFE) with cross-validation was applied to rank features according to their predictive importance. Boruta feature selection was performed using a random forest–based algorithm to identify features with statistically significant importance relative to shadow attributes. For LVQ, variable importance scores were calculated, and genes with importance values greater than 0.5 were retained. Genes were ranked based on feature importance derived from each algorithm. A gene was defined as a candidate hub gene if it was identified as an important feature by at least four of the eight machine learning algorithms, reflecting a consensus-based selection strategy. The term “hub gene” is used here to denote genes with high predictive relevance across multiple models rather than to imply definitive biological causality. The overlap and unique gene sets identified by different algorithms were visualized using an UpSet plot generated with the ‘UpSet’ R package.

### 2.6 Immune cell composition estimation using CIBERSORT

To characterize immune cell composition in peripheral blood following SCI, immune cell proportions were estimated using the CIBERSORT algorithm. CIBERSORT is a computational deconvolution approach that infers the relative proportions of 22 immune cell subtypes from bulk transcriptomic data based on a validated leukocyte gene signature matrix (24). In this study, the LM22 signature matrix was applied, and the analysis was performed using the ‘CIBERSORT’ R package with 100 permutations. Quantile normalization was enabled (QN = TRUE), as the input data consisted of normalized gene expression values rather than raw count data. For each sample, CIBERSORT generated relative immune cell proportion estimates along with associated deconvolution metrics. Only immune cell proportion estimates were retained for downstream analyses. Immune cell types with zero estimated abundance across all samples were excluded. The resulting immune cell composition profiles were compared between the SCI and HC groups. The distribution of immune cell proportions and pairwise correlations among immune cell subtypes were visualized using the ‘ggplot2’ and ‘ggcorrplot’ packages, respectively. Associations between hub gene expression levels and estimated immune cell proportions were evaluated using Spearman correlation analysis. Given that the analysis was based on peripheral blood transcriptomic data, the results were interpreted as estimates of immune cell composition rather than direct measurements of immune cell infiltration or tissue-level immune microenvironment.

### 2.7 Pathway associations in samples stratified by FCGR1A expression

To explore the biological functions associated with the hub gene, Gene Set Enrichment Analysis (GSEA) was performed based on the expression level of the hub gene. Samples were ranked according to hub gene expression, and enrichment analysis was conducted to identify pathways associated with high and low expression patterns. An adjusted *P*-value < 0.05 was considered statistically significant. The top five significantly enriched pathways for both upregulated and downregulated gene sets were selected based on normalized enrichment scores (NES).

### 2.8 Construction of the protein–drug interaction network

To identify potential drugs targeting the hub gene, protein–drug interaction analysis was performed using the NetworkAnalyst platform (https://www.networkanalyst.ca/) (25). Based on its regulatory role in SCI, the hub gene was used as the input to retrieve protein–drug interaction information derived from the DrugBank database. The resulting protein–drug interaction network was subsequently visualized using Cytoscape software (26).

### 2.9 Regulatory network analysis

To explore upstream regulatory mechanisms of the hub gene, a regulatory network integrating the hub gene, microRNAs (miRNAs), and transcription factors (TFs) was constructed using the NetworkAnalyst platform (https://www.networkanalyst.ca/) (25). The regulatory interaction data were obtained from the RegNetwork database. The resulting network was visualized and further refined using Cytoscape software (26).

### 2.10 Experimental animals

*In vivo* experiments were conducted to validate the expression of FCGR1A, which was identified by bioinformatic analysis as an upregulated NET-associated gene in SCI. Twelve adult female Sprague–Dawley rats (6–8 weeks old, 150–200 g) were obtained from the Laboratory Animal Center of Xi’an Jiaotong University (license number: SCXC (Shan) 2023–002). Animals were randomly assigned to either a sham group (n = 6) or an SCI group (n = 6). All experimental procedures were approved by the Animal Ethics Committee of Yan’an University and conducted in accordance with institutional guidelines. After randomization, rats were anesthetized by intraperitoneal injection of 3% sodium pentobarbital (40 mg/kg). A laminectomy was performed at the T10 vertebral level to expose the dorsal surface of the spinal cord. In the SCI group, spinal cord injury was induced using the HI-0400 percussion device (Precision Systems and Instrumentation, PSI) by delivering an impact force of 200 kdyn to the T10 spinal segment, as previously described (27). Rats in the sham group underwent laminectomy without impact injury, followed by wound closure. Postoperatively, all rats received intramuscular penicillin (50,000 U/kg/day) for three consecutive days. Daily nursing care was provided, and manual bladder expression was performed twice daily. Spinal cord tissue samples were collected 3 days after SCI induction.

### 2.11 Extraction of RNA and quantitative real-time PCR analysis

Total RNA was extracted from spinal cord tissue samples using the Simgen Ultra-Pure Total RNA Extraction Kit (5003050-50T, Simgen, China) according to the manufacturer’s instructions. Complementary DNA (cDNA) was synthesized using the PrimeScript™ RT Reagent Kit (Takara, Japan). Quantitative real-time PCR (qRT-PCR) was performed on an ABI 7500 Fast Real-Time PCR System (Thermo Fisher Scientific, USA). Relative mRNA expression levels were calculated using the 2^^−ΔΔ^Ct method, with β-actin serving as the internal control. Primer sequences are listed in **Table 1**.

**Table 1:** Sequences of primers used in this study.

Gene	Sequence (5′–3′)
*Fcgr1a*	Forward: AACGACTCTGCTACTTTGGGT
	Reverse: TGAGGTCCCTCACACAACAAA
β-actin	Forward: CCCATCTATGAGGGTTACGC
	Reverse: TTTAATGTCACGCACGATTTC

### 2.12 Immunofluorescence staining

Frozen spinal cord tissue sections (10 μm) were fixed and blocked with 5% normal goat serum at room temperature. The sections were then incubated overnight at 4 °C with a primary antibody against FCGR1A/CD64 (1:200, Bioss, China; bs-3511R). After washing to remove unbound primary antibodies, the sections were incubated with a fluorescently labeled secondary antibody (IgG, A23320, Abbkine, China) at room temperature in the dark. Nuclei were counterstained with DAPI (Beyotime, Shanghai, China) for 5 min after three final washes.

### 2.13 Statistical analysis

Statistical analyses were performed using R software (version 4.1.3) with appropriate packages, and experimental data were analyzed using GraphPad Prism 9.0. All data are presented as the mean ± standard error of the mean (SEM). Comparisons between two groups were conducted using a two-tailed Student’s t-test. A *P*-value < 0.05 was considered statistically significant, with significance levels indicated as *P* < 0.05 (*), *P* < 0.01 (**), and *P* < 0.001 (***).

## 3 Results

### 3.1 WGCNA identifies gene modules associated with SCI

**Figure 2:**
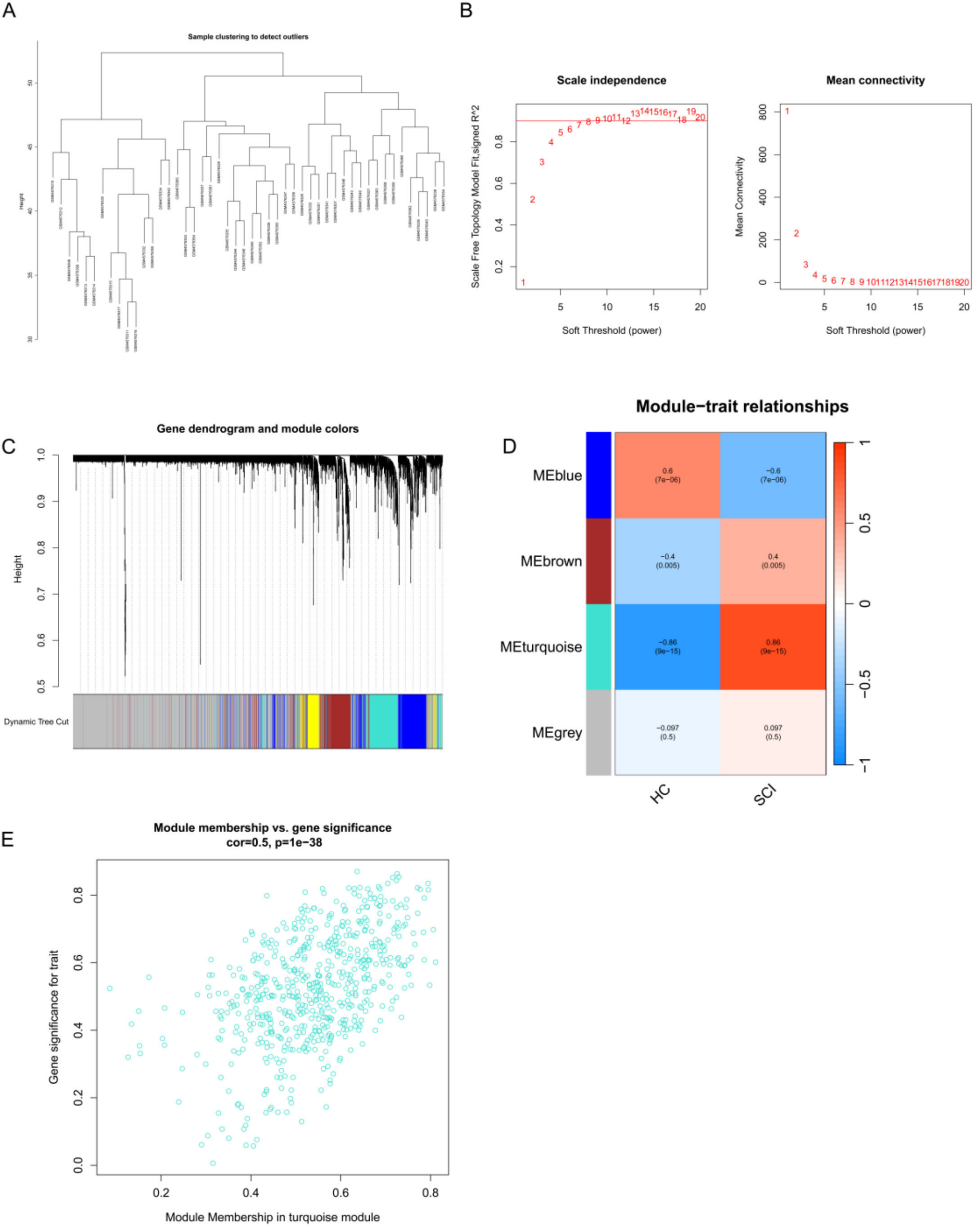
Identification of gene modules associated with SCI by WGCNA. **(A)** Hierarchical clustering dendrogram of samples from the GSE151371 dataset. **(B)** Analysis of scale-free topology fit index and mean connectivity for selecting the optimal soft-thresholding power. **(C)** Module clustering dendrogram, with different colors representing distinct gene modules. **(D)** Heatmap showing correlations between module eigengenes and clinical traits, including HC and SCI samples. **(E)** Scatter plot illustrating the relationship between MM and GS within the turquoise module.

To identify gene co-expression modules associated with SCI, WGCNA was performed using the GSE151371 dataset. Hierarchical clustering was conducted to evaluate sample clustering and potential outliers. The resulting dendrogram showed that samples clustered according to experimental groups, with no obvious outliers observed (**Figure 2A**). A soft-thresholding power of 9 was selected to construct a scale-free network (**Figure 2B**). Four gene modules were subsequently identified. Among them, the turquoise module exhibited the strongest positive correlation with SCI (cor = 0.86, *P* = 9 × 10^-15^) (**Figures 2C, D**). Given its high association with SCI, the turquoise module was selected for further analyses. To further assess the relevance of the turquoise module, the relationship between gene significance (GS) and module membership (MM) was examined. A strong positive correlation was observed between GS and MM values (cor = 0.50, *P* < 1 × 10-^38^), indicating that genes highly connected within this module tended to be more strongly associated with SCI. Based on this analysis, 591 genes within the turquoise module were retained for subsequent analyses (**Figure 2E**).

### 3.2 Expression patterns of NETs-related differentially expressed genes in SCI

**Figure 3:**
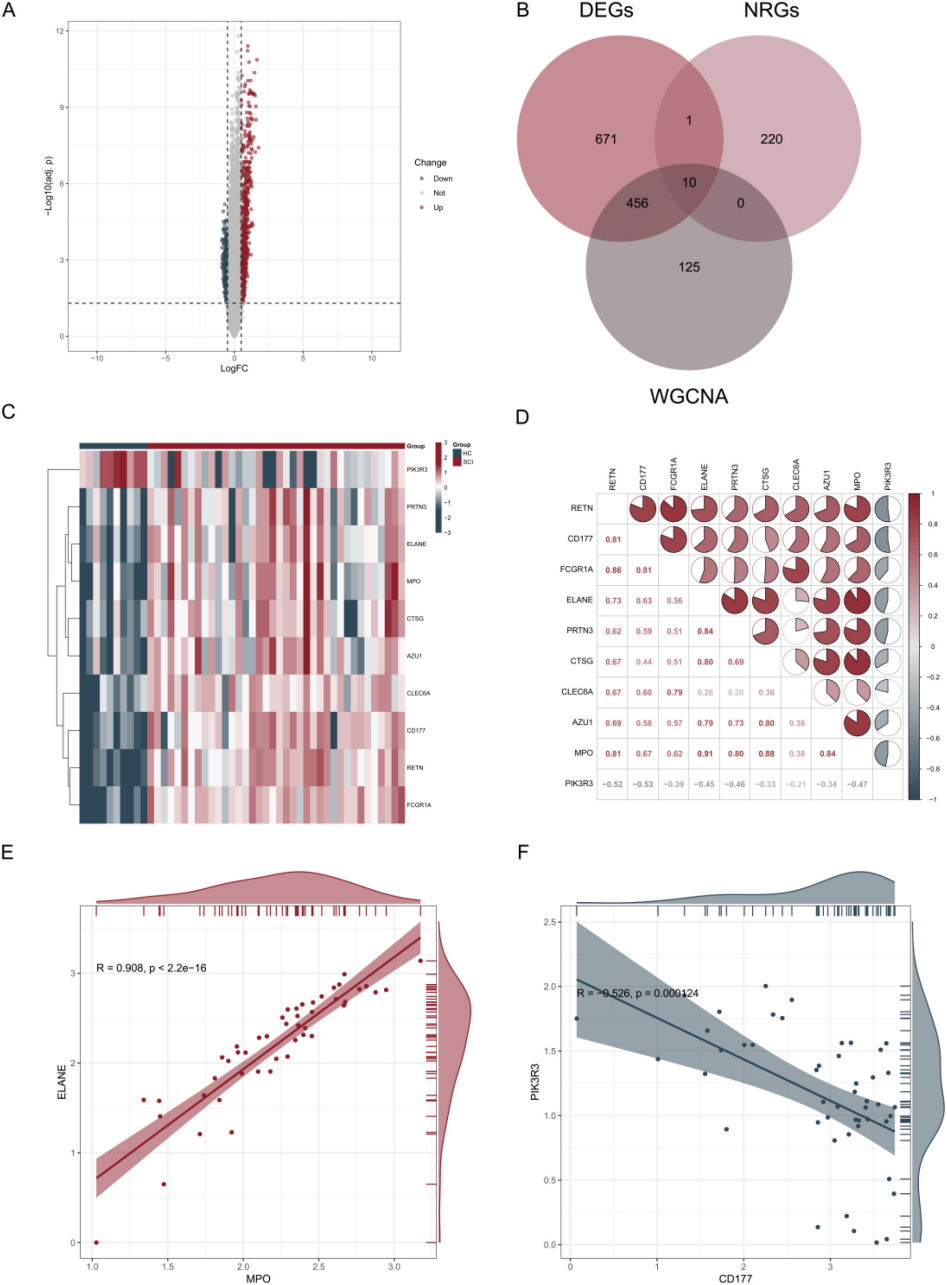
Expression and correlation analysis of NET-related differentially expressed genes in SCI. **(A)** Volcano plot showing differentially expressed genes between SCI and HC samples in the GSE151371 dataset. **(B)** Venn diagram illustrating the overlap among NRGs, DEGs, and genes from the turquoise WGCNA module, resulting in the identification of DE-NRGs. **(C)** Heatmap displaying the expression patterns of DE-NRGs across all samples. **(D)** Pearson correlation matrix of pairwise gene expression levels among DE-NRGs. **(E, F)** Scatter plots showing representative gene expression correlations, including a positive correlation between MPO and ELANE and a negative correlation between CD177 and PIK3R3.

To identify DEGs associated with SCI, transcriptomic data from the GSE151371 dataset, comprising 38 SCI samples and 10 HCs, were analyzed. Using thresholds of |log_2_FC| > 0.5 and an adjusted *P* < 0.05, a total of 1,138 DEGs were identified, including 707 upregulated and 431 downregulated genes (**Figure 3A**). To identify NET-related genes associated with SCI, an intersection analysis was performed among the DEGs, genes within the turquoise module, and a predefined set of 231 NET-related genes. This analysis resulted in the identification of 10 DE-NRGs (**Figure 3B**). The expression patterns of these 10 DE-NRGs across all samples are shown in a heatmap (**Figure 3C**). Correlation analysis revealed significant expression correlations among the 10 DE-NRGs (**Figure 3D**). The strongest positive correlation was observed between ELANE and MPO (r = 0.908; **Figure 3E**), while a notable negative correlation was detected between CD177 and PIK3R3 (r = −0.526; **Figure 3F**). These results indicate that NET-related genes exhibit coordinated expression patterns in SCI, suggesting potential regulatory relationships that warrant further investigation.

### 3.3 GO and KEGG enrichment analyses of DE-NRGs

**Figure 4:**
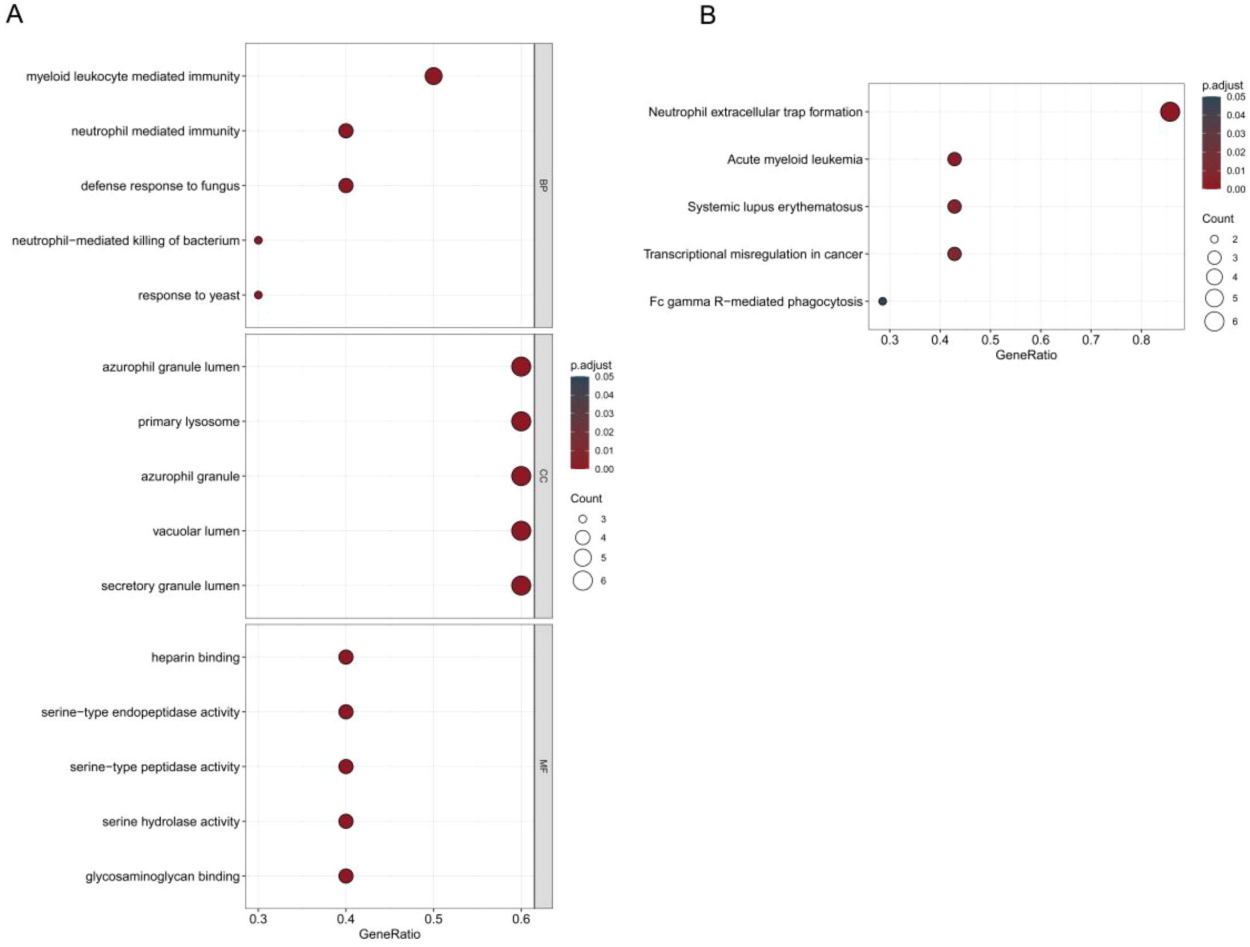
GO and KEGG pathway enrichment analysis of DE-NRGs. **(A)** GO enrichment analysis of DE-NRGs, visualized as a bubble plot. **(B)** KEGG pathway enrichment analysis of DE-NRGs, visualized as a bubble plot.

To explore the biological processes and pathways associated with the DE-NRGs, GO and KEGG enrichment analyses were performed. GO enrichment analysis indicated that DE-NRGs were significantly associated with immune-related biological processes, particularly those involving myeloid cells, neutrophil-mediated responses, granule organization, and extracellular signaling (**Figure 4A**). KEGG pathway analysis further revealed that DE-NRGs were significantly enriched in pathways related to neutrophil extracellular trap formation (**Figure 4B**).

### 3.4 Prioritizing key genes mediating NETs–SCI crosstalk using machine learning

**Figure 5:**
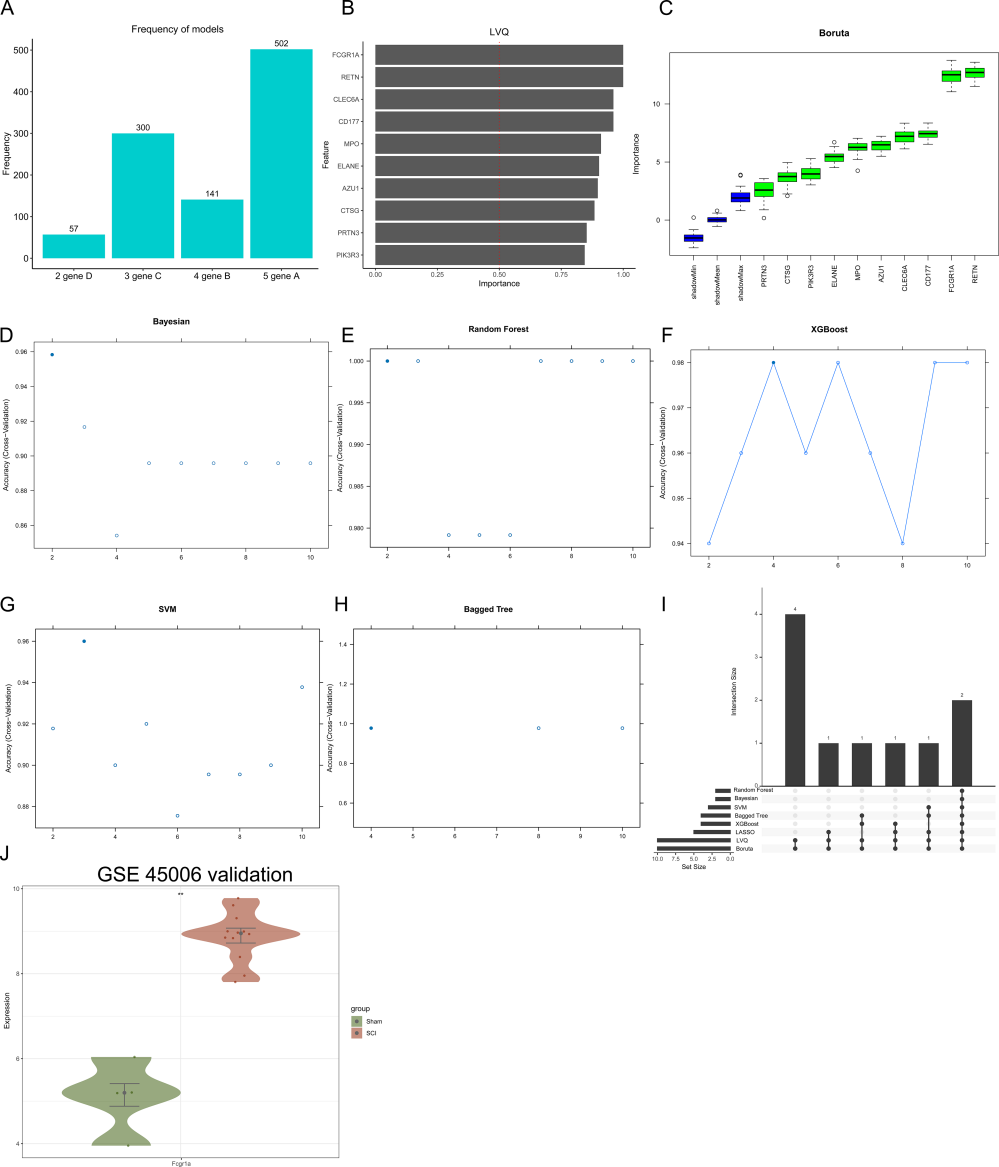
Prioritization of NET-related candidate genes associated with SCI using machine learning. Feature selection results obtained from multiple machine learning algorithms applied to the GSE151371 dataset. **(A)** Lasso logistic regression (1,000 iterations). **(B)** LVQ. **(C)** Boruta. **(D)** Bayesian model. **(E)** RF. **(F)** XGBoost. **(G)** SVM. **(H)** Bagged Trees. **(I)** Overlap of candidate genes identified by all eight machine learning algorithms. **(J)** Validation of candidate gene expression in the independent GSE45006 dataset. **P < 0.01.

To identify key genes involved in the pathogenesis of SCI, we applied eight machine learning algorithms to ten DE-NRGs derived from the GSE151371 dataset. These algorithms included Lasso logistic regression (1,000 iterations), LVQ, Boruta, Bayesian analysis, RF, XGBoost, SVM, and Bagged Trees (**Figures 5A–H**). Intersection analysis across all eight models identified three consensus genes—CLEC6A, FCGR1A, and RETN—as being strongly associated with SCI pathogenesis (**Figure 5I**). To further validate these findings, the expression levels of the three candidate genes were assessed in an independent dataset (GSE45006). Among them, only FCGR1A exhibited significant differential expression, thereby confirming its association with SCI (**Figure 5J**). Collectively, these results suggest that FCGR1A may serve as a marker associated with NETs involvement in the pathogenesis of SCI.

### 3.5 Analysis of immune cell composition estimated by CIBERSORT

**Figure 6:**
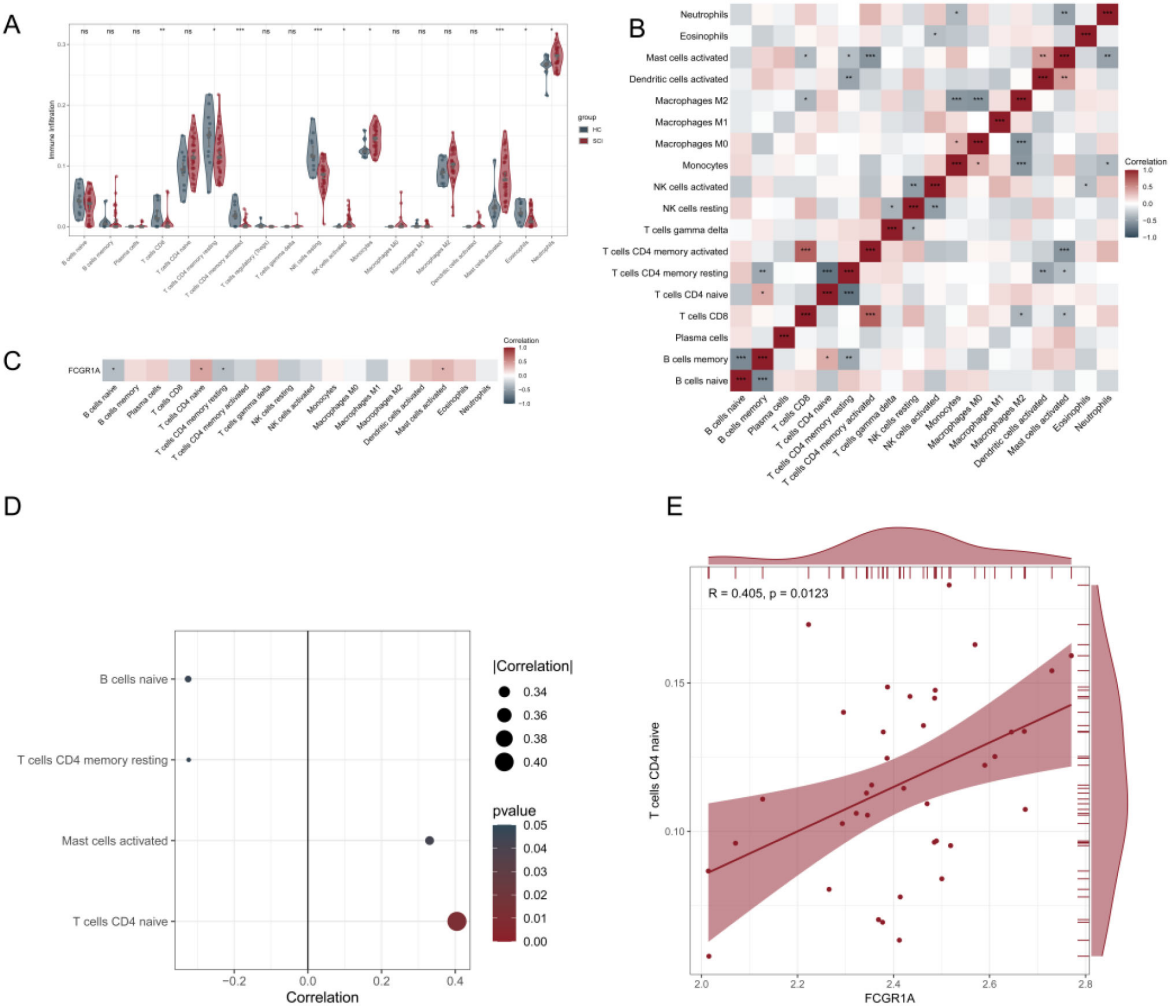
Immune cell composition analysis associated with the hub gene. **(A)** Violin plots illustrate differences in immune cell composition between the SCI and HC groups. **(B)** Correlation matrix showing the relationships among the proportions of different immune cell subtypes, with color intensity representing the Pearson correlation coefficient. **(C)** Pearson correlation analysis between hub gene expression and immune cell composition in SCI samples. **(D)** Lollipop charts illustrate the correlations between hub gene expression and the abundance of immune cell subtypes. **(E)** Scatter plot showing the correlation between FCGR1A expression and the proportion of naïve CD4 T cells in SCI samples. *P < 0.05, **P < 0.01, ***P < 0.001.

To explore differences in immune cell composition between groups, immune cell proportions were estimated using the CIBERSORT algorithm. Compared with healthy controls, SCI samples exhibited altered immune cell composition profiles, characterized by increased estimated proportions of activated NK cells, monocytes, activated mast cells, and neutrophils, along with decreased proportions of CD8 T cells, resting and activated CD4 memory T cells, resting NK cells, and eosinophils (**Figure 6A**). Correlation analysis revealed coordinated associations among immune cell populations. Activated CD4 memory T cells were positively correlated with CD8 T cells and negatively correlated with activated mast cells. In addition, activated mast cells showed a positive correlation with activated dendritic cells, indicating patterns of immune cell co-variation within SCI samples (**Figure 6B**). Further analysis demonstrated that FCGR1A expression levels in SCI samples were positively correlated with the estimated abundance of activated mast cells and naïve CD4 T cells, whereas negative correlations were observed with naïve B cells and resting memory CD4 T cells (**Figure 6C**). Lollipop charts were used to visualize these associations, among which naïve CD4 T cells showed the strongest correlation with FCGR1A expression (**Figure 6D**). Consistently, scatter plot analysis confirmed a positive correlation between FCGR1A expression and the estimated proportion of naïve CD4 T cells in SCI samples (R = 0.405, *P* = 0.0123) (**Figure 6E**). Collectively, these results indicate that FCGR1A expression is associated with distinct immune cell composition patterns in SCI, as inferred from CIBERSORT-based estimation, highlighting correlations that warrant further experimental validation.

### 3.6 Pathway and functional annotation associated with FCGR1A expression

**Figure 7:**
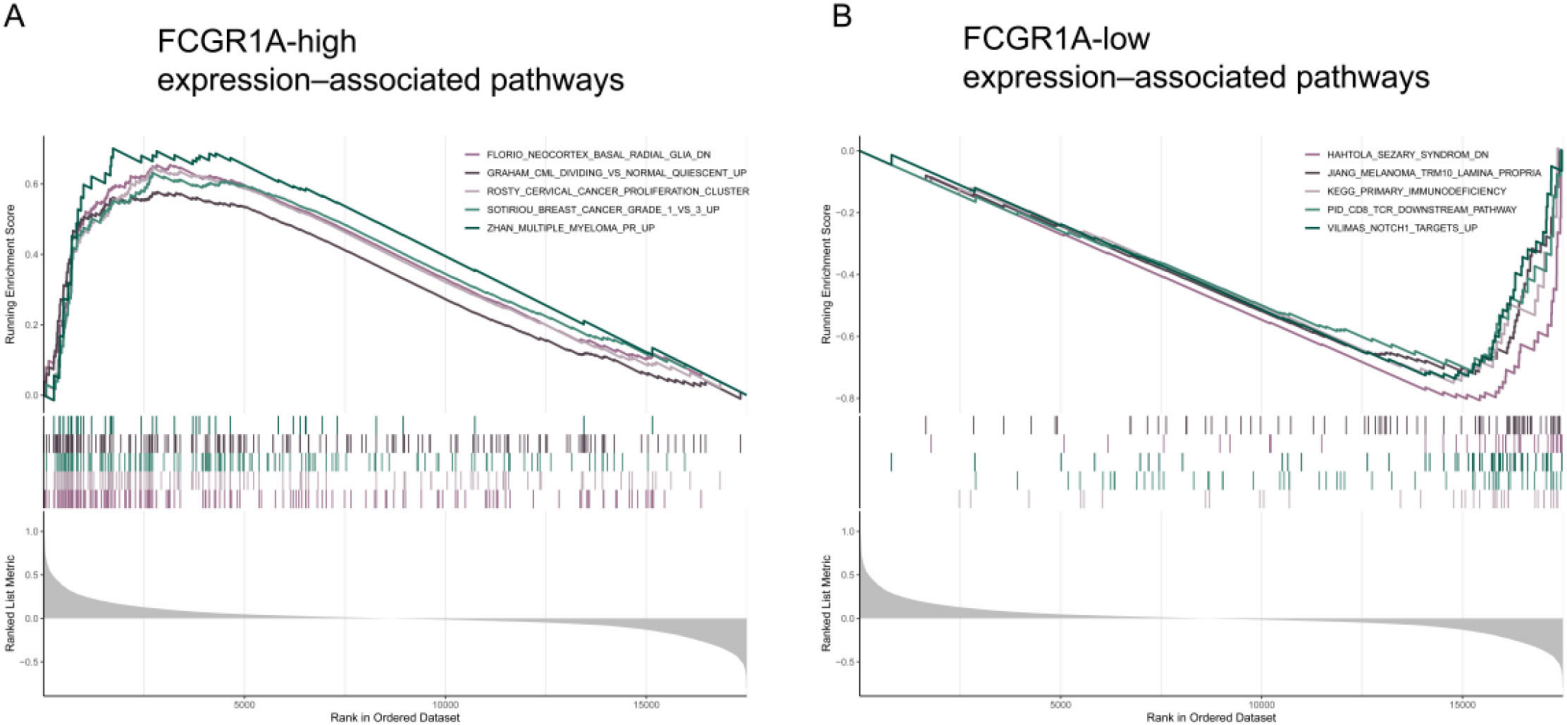
Pathway associations correlated with FCGR1A expression. **(A)** The five most significantly enriched pathways positively associated with high FCGR1A expression. **(B)** The five most significantly enriched pathways negatively associated with low FCGR1A expression. Pathway enrichment was performed using a correlation-based approach stratified by FCGR1A expression and does not imply causal or mechanistic relationships.

To explore gene expression patterns associated with the hub gene, a correlation-based pathway association analysis stratified by FCGR1A expression was performed. As shown in **Figure 7A**, higher FCGR1A expression was associated with enrichment of gene sets annotated in cancer-related pathways, including signatures related to cervical cancer, breast cancer, leukemia, and multiple myeloma. These gene sets largely reflect shared inflammatory, immune, and cell proliferation–related signaling programs rather than cancer-specific biological processes. In contrast, lower FCGR1A expression was associated with enrichment of gene sets related to neurodevelopmental and stem cell–associated programs, as well as immune regulatory pathways, including those related to T-cell function and Notch1 target gene signatures (**Figure 7B**). Together, these results demonstrate distinct gene expression patterns associated with differential FCGR1A expression levels in SCI.

### 3.7 Molecular regulatory network and drug prediction

**Figure 8:**
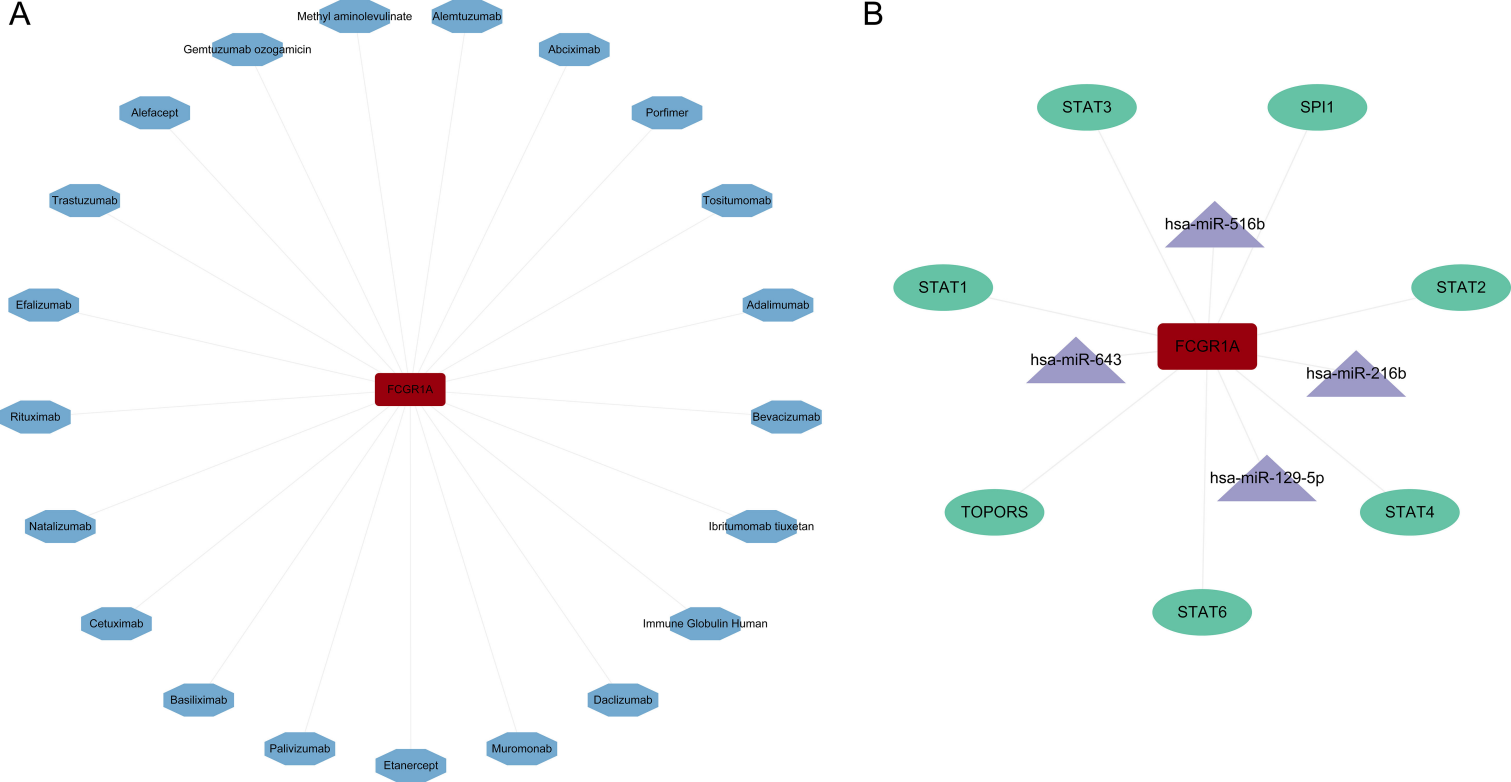
Construction of the drug–protein interaction network and regulatory network. **(A)** Drug–protein interaction network. In the network, the hub protein is represented by a red rectangle, while predicted drugs and molecular compounds are depicted as blue hexagons. **(B)** miRNA–hub gene–TF regulatory network constructed using Cytoscape. The hub gene is represented by a red rectangle, miRNAs by purple triangles, and TFs by green ellipses.

Next, a drug–protein interaction network centered on the hub gene was constructed to identify potential therapeutic agents (**Figure 8A**). To further explore the transcriptional and post-transcriptional regulatory mechanisms of the hub gene, bioinformatics analyses were conducted to predict upstream TFs and targeting miRNAs. The interaction network analysis identified a total of seven TFs and four miRNAs associated with the hub gene (**Figure 8B**). These findings suggest that the hub gene is regulated by a complex network involving multiple TFs and miRNAs.

### 3.8 Experimental validation of FCGR1A *in vivo*

**Figure 9:**
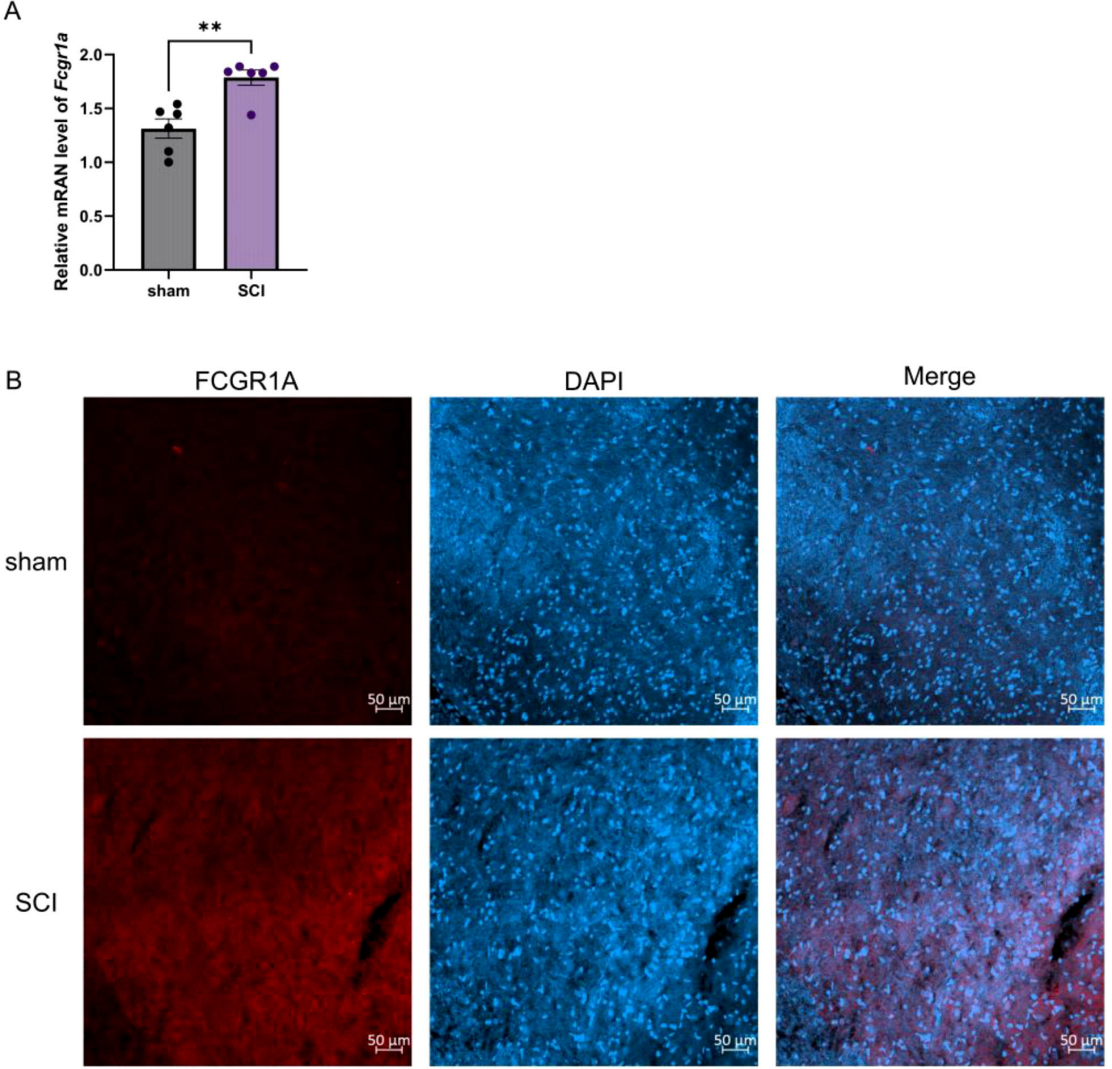
Validation of hub gene expression in an established animal model of the disease. **(A)** Comparison of hub gene mRNA levels between the sham and SCI groups in rats. **(B)** IF staining of the hub gene protein in rat spinal cord tissue. **P < 0.01.

To provide biological validation for the bioinformatic findings, FCGR1A expression was examined in spinal cord tissues following SCI. Quantitative real-time PCR analysis demonstrated that FCGR1A mRNA expression was significantly increased in the SCI group compared with the sham group (**Figure 9A**). Consistently, immunofluorescence staining revealed elevated FCGR1A expression in spinal cord tissues from SCI rats relative to sham controls (**Figure 9B**).

## 4 Discussion

Traumatic SCI represents a catastrophic neurological event characterized by profound and often irreversible neurological deficits and permanent tissue damage. The pathophysiology of SCI involves an immediate primary injury caused by mechanical trauma, followed by a complex and evolving secondary injury cascade, in which neuroinflammation emerges as a central pathogenic feature (28). Among the myriad inflammatory responses following SCI, NETs have emerged as important contributors to secondary damage. NETs are extracellular, web-like structures released by activated neutrophils, primarily composed of DNA fibers decorated with histones and cytotoxic granular proteins (29). Although NETs play an essential role in host defense against pathogens, excessive or dysregulated NET formation in the context of SCI exacerbates secondary tissue injury. NETs act as potent amplifiers of the inflammatory cascade by directly activating immune cells and promoting the release of key pro-inflammatory mediators, including cytokines and chemokines. Beyond their pro-inflammatory effects, NETs can also induce secondary ischemia by causing vascular occlusion, thereby critically reducing blood flow and further aggravating tissue destruction in the injured spinal cord (30). In this study, we sought to elucidate key NET-associated genes involved in SCI through an integrative multi-omics and machine learning framework, with subsequent *in vivo* validation. Our findings advance the understanding of the pathophysiology and molecular features associated with SCI and identify NET-related candidate genes that may warrant further mechanistic investigation.

WGCNA identified a module comprising 591 genes that were significantly associated with SCI. Differential expression analysis of the GSE151371 dataset identified a substantial number of dysregulated genes in SCI. Intersection of the DEGs, WGCNA module genes, and NRGs yielded 10 DE-NRGs for further investigation. Functional enrichment analyses revealed that these DE-NRGs were significantly enriched in neutrophil-mediated immune processes and the neutrophil extracellular trap formation pathway, as demonstrated by both GO and KEGG analyses. It should be noted that the GO and KEGG enrichment analyses were performed on a relatively small set of intersected genes derived from differential expression, WGCNA modules, and a predefined NET-related gene signature. As such, these enrichment results are exploratory in nature and may be subject to limited statistical power and potential circularity. Therefore, the enrichment findings should be interpreted with caution and primarily viewed as supportive evidence of NET-associated biological relevance rather than as unbiased pathway discovery. Following machine learning–based prioritization, three candidate hub genes were identified. However, validation using an independent dataset (GSE45006) revealed that only FCGR1A exhibited consistent and significant differential expression, supporting its robustness in the context of SCI. Importantly, the machine learning analyses were conducted within a hypothesis-driven framework and were intended to facilitate candidate prioritization rather than to serve as an unbiased genome-wide discovery approach.

We subsequently performed an immune cell composition analysis to characterize systemic immune alterations in SCI and to assess their association with FCGR1A expression. Among all evaluated immune cell subtypes, FCGR1A expression showed the strongest correlation with naïve CD4 T cell proportions. As this analysis was based on peripheral blood transcriptomic data, the estimated immune cell proportions reflect systemic immune states rather than direct immune cell infiltration at the injury site. Although SCI is known to induce pronounced systemic immune alterations that may be linked to local neuroinflammatory processes, peripheral immune profiles may not fully capture the complex cellular environment within the injured spinal cord and should therefore be interpreted with caution.

Correlation-based pathway association analysis indicated that FCGR1A expression was associated with gene sets commonly annotated in cancer-related pathways, which largely reflect shared inflammatory, immune, and cell proliferation–related signaling programs rather than cancer-specific mechanisms. Additionally, FCGR1A expression exhibited negative correlations with certain gene sets annotated to regulatory biological processes. Importantly, these findings do not establish causal or mechanistic relationships but instead describe pathway-level associations linked to FCGR1A expression patterns in the context of SCI. To further characterize the potential regulatory context of FCGR1A, we performed in silico predictive analyses to identify upstream miRNAs and TFs, as well as compounds predicted to interact with FCGR1A-related protein networks. These analyses were conducted for hypothesis-generation purposes only, aiming to provide a preliminary framework for future experimental investigation of FCGR1A-associated molecular regulation in SCI, including its possible relevance to NET-related processes. Accordingly, all predicted miRNAs, TFs, and compounds should be interpreted as computationally inferred candidates requiring rigorous experimental validation, rather than as evidence of confirmed regulatory mechanisms or therapeutic strategies for SCI.

The protein product of the FCGR1A gene is CD64 (FcγR1A), a high-affinity Fc receptor for immunoglobulin G (IgG). Its expression is largely restricted to cells of the myeloid lineage, including macrophages, monocytes, and dendritic cells, where it serves as an important mediator of immune effector functions (31). Upon binding to IgG-containing immune complexes, CD64 participates in antibody-dependent cellular phagocytosis and cytotoxic responses (32). Under physiological conditions, CD64 is constitutively expressed on monocytes and macrophages. During infection or inflammatory states, its expression can be markedly upregulated, particularly on neutrophils, where surface levels may increase rapidly (33). In transgenic mouse models, neutrophils have been shown to contribute substantially to CD64-associated allergic inflammatory responses (34). In macrophages, CD64 expression is preferentially increased in classically activated (M1) phenotypes and reduced in alternatively activated (M2) phenotypes, making it a well-recognized marker of pro-inflammatory macrophage polarization in chronic inflammatory settings (35, 36). Functionally, FCGR1A engages antigen–antibody complexes and is involved in immune signaling processes that support immune cell activation and inflammatory mediator release (37). Consistent with this role, FCGR1A has been implicated in the pathogenesis of several chronic inflammatory and autoimmune diseases, including rheumatoid arthritis and systemic lupus erythematosus (38, 39). Emerging evidence also suggests that FCGR1A expression is associated with immune regulatory features in colorectal cancer (40). However, to date, the involvement of FCGR1A in the context of SCI has not been previously reported.

In the present study, *in vivo* experiments were performed to biologically validate the bioinformatic prediction that FCGR1A is an upregulated NET-associated gene in SCI. Consistent with the computational analyses, FCGR1A expression was significantly increased in spinal cord tissues following SCI, supporting its association with NET-related inflammatory signatures in this condition. These findings suggest that FCGR1A may serve as a potential NET-associated biomarker in SCI. However, the precise functional roles and molecular mechanisms by which FCGR1A contributes to NET formation and SCI pathophysiology remain to be fully elucidated. Further investigation into NET-related signaling pathways may provide new insights into immune-mediated mechanisms of SCI and identify potential therapeutic targets.

Despite these encouraging findings, several limitations of this study should be acknowledged. First, although FCGR1A was identified as a hub gene associated with NET-related immune responses in SCI, additional experiments are required to elucidate the precise molecular mechanisms through which it may regulate NET formation and influence SCI pathology. A deeper mechanistic understanding will be essential for advancing the therapeutic exploration of NETs in SCI. Second, a key limitation lies in the mismatch between the discovery and validation datasets. Gene discovery was based on transcriptomic data derived from human peripheral blood, whereas experimental validation was performed using spinal cord tissue from a rat SCI model. Given the differences in species and tissue-specific microenvironments, NET-related gene expression patterns in peripheral blood may not be directly comparable to those in spinal cord tissue. Therefore, our findings should be interpreted with appropriate caution, and further validation using tissue-matched and species-consistent datasets will be necessary to strengthen translational relevance. Third, the relatively small number of healthy control samples in the discovery dataset (n = 10) represents an additional limitation. Although integrative approaches such as WGCNA and machine learning were applied to improve robustness, limited sample size may affect network stability, feature selection, and hub gene identification, potentially leading to overinterpretation or redundancy. Accordingly, the present results should be regarded as hypothesis-generating rather than definitive functional evidence. Fourth, although FCGR1A expression was validated at both the mRNA and protein levels *in vivo*, direct functional manipulation of this gene, such as knockdown or overexpression, was not performed. Future studies incorporating gene perturbation approaches will be required to establish causal relationships and to clarify the specific roles of FCGR1A in NET formation and SCI progression. Finally, validation in larger and more diverse cohorts is warranted to enhance the robustness of these findings. In addition, investigating the temporal dynamics of FCGR1A expression across different stages of SCI and defining its precise contribution to disease pathogenesis will be critical steps toward evaluating its potential clinical and therapeutic applicability.

## 5 Conclusion

In conclusion, this study identifies FCGR1A as an upregulated gene associated with NET-related inflammatory signatures in SCI through integrative bioinformatic analyses and *in vivo* expression validation. While FCGR1A was highlighted as a consistently dysregulated candidate within the analyzed gene networks, the present findings do not establish causal or functional roles for this gene in SCI.

Rather, our results provide a hypothesis-generating framework that may inform future experimental studies aimed at elucidating the mechanistic and functional relevance of FCGR1A and NET-associated immune processes in SCI. By integrating transcriptomic analyses with machine learning–based prioritization, this work offers a data-driven resource to guide subsequent mechanistic investigations rather than definitive conclusions regarding disease regulation or therapeutic intervention.

## Data Availability

The datasets presented in this study can be found in online repositories. The names of the repository/repositories and accession number(s) can be found in the article/**Supplementary Material**.
